# Salivary Neurotrophins Brain-Derived Neurotrophic Factor and Nerve Growth Factor Associated with Childhood Obesity: A Multiplex Magnetic Luminescence Analysis

**DOI:** 10.3390/diagnostics12051130

**Published:** 2022-05-03

**Authors:** Vaithinathan Selvaraju, Jeganathan R. Babu, Thangiah Geetha

**Affiliations:** 1 Department of Nutrition, Dietetics and Hospitality Management, Auburn University, Auburn, AL 36849, USA; vzs0041@auburn.edu (V.S.); jeganrb@auburn.edu (J.R.B.); 2 Boshell Diabetes and Metabolic Diseases Research Program, Auburn University, Auburn, AL 36849, USA

**Keywords:** brain-derived neurotrophic factor (BDNF), β-nerve growth factor (β-NGF), childhood obesity, saliva, biomarker

## Abstract

Obesity is linked with higher inflammatory markers and is characterized by chronic low-grade inflammation. Neurotrophins brain-derived neurotrophic factor (BDNF) and β-nerve growth factor (β-NGF), in addition to their neuronal functions, act on several immune cells and have been recently designated as metabokines due to their regulatory role in energy homeostasis and food intake. The current study evaluates the salivary BDNF and β-NGF and their association with anthropometric measurement, blood pressure, and salivary insulin in children. Anthropometric measurements and saliva samples were obtained from 76 children, aged 6–10 years. Multiplex analysis was carried out for the salivary analysis of BDNF, NGF, and insulin by human magnetic Luminex performance assay. Statistical analysis was performed to analyze the best fit diagnostic value for biomarkers and the relationship of the neurotrophic levels of BDNF and NGF with obesity measures and blood pressure. Salivary BDNF and β-NGF showed a significantly higher concentration in obese children than normal-weight children. Both neurotrophins are positively associated with obesity anthropometric measures, blood pressure, and salivary insulin. Multinominal regression analysis reported a significant association between salivary BDNF, β-NGF, insulin, and systolic pressure adjusted for age, gender, income, and maternal education. The salivary concentration of BDNF and NGF was higher in obese children, and it is positively associated with anthropometric measures, suggesting that neurotrophins can be used as a non-invasive predictor of obesity-related complications in children.

## 1. Introduction

The global epidemiologic of metabolic disorders is associated with obesity, which is a primary threat to human health in the current century, reducing human lifespan. If the current secular trend continues with the worldwide adult population, an expected overweight of 38% and an additional 20% of the obese population might be reached by 2030 [1,2]. The United States of America’s prevalence of obesity has risen by 18.5% since the early 1990s. Over the past few decades, obesity has been a major problem among children and has affected approximately 13.7 million children and teenagers during 2015 and 2016 [3]. Overweight and obesity prevalence in early life elevates the risk of developing obesity-associated health issues in adulthood. Early life detection of the metabolic risk factor could help protect children from the widespread consequences of obesity-related diseases.

Biomarkers for diagnosing disease conditions are analyzed in many body fluids, such as serum, plasma, urine, and cerebrospinal fluid [2,4]. Recent investigations have revealed that disease biomarkers for diagnosing inflammation, obesity, insulin resistance, and other metabolic disorder can be identified in saliva [5,6,7,8]. The significant benefit of saliva is that it is easy to collect and does not require specialized equipment. It is a non-invasive and relatively stress-free possible alternative diagnostic agent in children. The contents of saliva are regulated by the systemic alterations that allow the determination of salivary metabolites and hormones used as a diagnostic means [9]. Serum biomarkers positively correlate with salivary biomarkers, which can detect diabetes, inflammation, and metabolic syndrome [10,11].

Brain-derived neurotrophic factor (BDNF) and nerve growth factor (NGF) are neurotrophins that show a pivotal role in the development and plasticity of the central nervous system [12]. Neurotrophins are involved in the development and progression of inflammatory and immune diseases. Obesity is the state of chronic low-grade inflammation; both murine and human adipose tissues release different inflammatory mediators, including NGF. The circulating neurotrophins play an essential role in the pathogenesis of metabolic syndrome [13]. Growing evidence reveals that BDNF is an essential factor in regulating food intake [14,15,16]. Previous studies confirm that BDNF is also necessary for body weight control and energy homeostasis. Reports show that obese and type 2 diabetic individuals exert a low circulating BDNF in their bodies [17] and show an inverse relationship between BDNF concentration and BMI in children and adults [18]. Krabbe et al. show that BDNF is also necessary for body weight control and energy homeostasis [17]. Recent studies reported conflicting data that modified the association with low BDNF levels in overweight participants [19,20,21]. The meta-analysis conducted by Sandrini et al. shows the circulating levels of BDNF in obese patients are equal to control participants. There was no difference observed between normal and obese patients [22]. Interestingly, Lee et al. demonstrated that weight reduction decreased the BDNF area under the curve (AUC) index, and serum BDNF AUC index was associated with obesity [23].

Children with obesity are at a higher risk of heart disease in adulthood. Obese children have higher systolic and diastolic blood pressure due to dyslipidemia and insulin resistance. Neurotrophins regulate glucose and energy metabolism in type 2 diabetes patients [24]. Salivary NGF was demonstrated to increase painful diseases such as burning mouth syndrome and chronic migraines [25,26] or stress [27]. This study aimed to measure neurotrophins (BDNF, NGF) and insulin in the saliva of normal-weight and overweight/obese children. In addition, the correlation of BDNF and NGF with salivary insulin, blood pressure, and obesity measures were evaluated. Our study provides a new sensitive method to measure neurotrophic factors in saliva as a great value to early prediction of obesity-related complications.

## 2. Materials and Methods

### 2.1. Study Participants

Forty normal-weight (NW), twenty overweight (OW), and sixteen obese (OB) children aged 6–10 years participated in the present work from Lee and Macon counties, AL. Auburn University IRB approval was obtained, and consent from parents and children was collected before recruitment to the study. Before the study, a phone survey was conducted to identify the children’s history of diabetes or cardiovascular disease from the parents to exclude from the analysis [28].

### 2.2. Anthropometric and Blood Pressure Measurements

On the day of sample collection, anthropometric measurements such as body weight, height, and waist circumference were taken as per the World Health Organization (WHO) recommended procedure. Briefly, the body weight and height of the children were recorded with the help of a Tanita digital scale attached to a stadiometer with minimal clothing [28]. The participant’s resting blood pressure (BP) and heart rate were recorded in a seated position using an automated BP apparatus (Omron blood pressure monitor). NW, OW, and OB groups were categorized based on the CDC growth chart [29]. The participant’s Body Mass Index (BMI) z-score was derived from the BMI, which was derived from the children’s weight and height. The relative weight was adjusted for child age and gender for BMI z-score calculation using the growth reference 2017 SPSS macro [30]. Waist circumference (WC) z-score, and waist-to-height ratio (WHtR) z-score were calculated using the R macro package [31].

### 2.3. Measurement of Salivary BDNF, NGF, and Insulin by Multiplex Analysis

The participants were requested to avoid any food or drink 30 min before obtaining the sample. The samples were collected using a saliva collection kit and stored at −80 °C until further analysis. The samples for multiplex analysis were centrifuged at 2800 rpm, 4 °C for 20 min to remove unwanted particulates [28]. Human BDNF and NGF were measured with a human premixed magnetic Luminex assay kit (catalog # LXSAHM, R&D systems, Minneapolis, MN, USA). The assay was carried out as per the manufacturer’s protocol. Standards and saliva samples (1:2 dilution) were briefly prepared using calibrator diluent RD6-52. The assay was performed with duplicate standards and samples (50 µL), along with premixed Luminex beads added to the plate and incubated for 2 h at room temperature (RT) with shaking (800 ± 50 rpm). The plate was washed three times with a wash buffer using a Bio-Plex handheld magnetic plate holder and then incubated with 50 µL diluted biotin-antibody cocktail for 1 h at RT on a shaker. Plates were washed with wash buffer and incubated with streptavidin-PE for 30 min at RT with shaking. The plate was washed three times and read using a Bioplex-3D analyzer. The details of the Multiplex measurement of insulin have been described in our previous study [7].

### 2.4. Statistical Analysis

**Figure 1:**
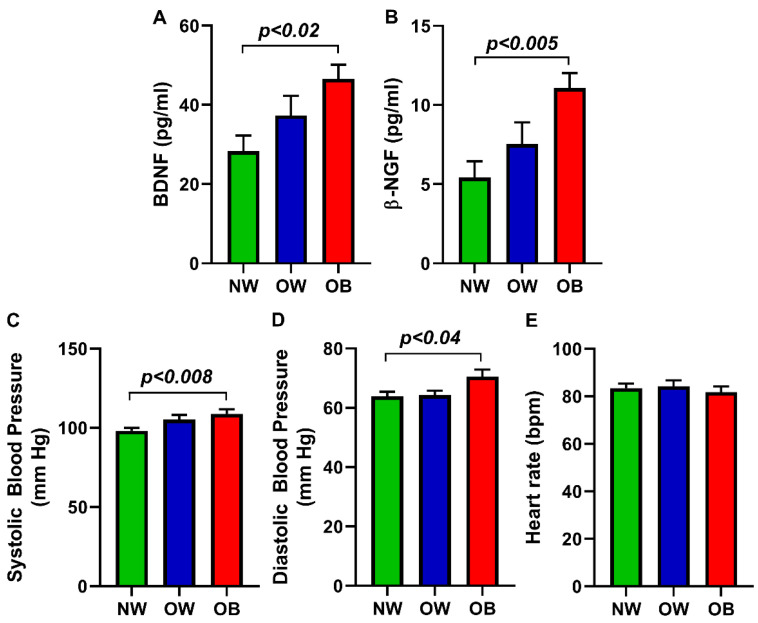
Bar graph shows the neurotrophin and pressure levels in the NW, OW, and OB groups. (**A**) BDNF, (**B**) NGF, (**C**) systolic pressure, (**D**) diastolic blood pressure, and (**E**) heart rate.

The results are expressed as the mean and standard error of the mean (SEM) in the bar graph. A one-way ANOVA test was performed by GraphPad prism (5.0, GraphPad Software, San Diego, CA, USA) for a three-group comparison. Tukey’s post-hoc test was carried out to show the difference between individual groups. Statistical significance between groups was provided on the graph, and the bar graphs without *p*-value are not significant at *p* < 0.05 levels (Figure 1). Data transformation was carried out before processing for further analysis for skewed data with a natural logarithm by SPSS (version 24, IBM, Armonk, NY, USA). The Receiver Operating Characteristic (ROC) curve analysis was performed to determine the diagnostic accuracy of BDNF and NGF. The area under the curve (AUC) was calculated to distinguish the high and low levels of diagnostic biomarkers. Multinomial regression analysis was carried out between parameters to determine the significantly affected biomarkers. The power analyses for the sample size have been described in Selvaraju et al. [7].

## 3. Results

Anthropometrics are quantitative body measures applied to evaluate growth, development, and health parameters. In the current study, participants’ anthropometric measurements were recorded. The obesity measurements showed a significant increase in the OW and OB groups compared to NW. The present study continues our previous study, and these results are shown in Table S1 of Supplementary Material [7].

The expression of salivary BDNF, the second member of the neurotrophic factors’ family, showed a significant increase in the OB (46.55 ± 3.63 pg/mL; *p* < 0.02) group compared to the NW group. The OW (37.32 ± 4.95 pg/mL) group showed an increased expression of BDNF, but there was no significant difference compared to the NW group (28.33 ± 3.94 pg/mL). Similarly, the best-known neurotrophin NGF, also recently identified as adipokines associated with obesity, exhibited a significant elevation in the OB group (11.08 ± 0.94 pg/mL; *p* < 0.005) in comparison with NW participants. NGF was higher in the OW group (7.53 ± 1.37 pg/mL) compared to the NW group (5.41 ± 1.03 pg/mL) but did not show significance. The results are expressed in the bar graph in Figure 1. In addition, we assessed the participant’s blood pressure and heart rate. OB group participants expressed significantly higher systolic (108.81 ± 2.85; *p* < 0.008) and diastolic (70.50 ± 2.40; *p* < 0.04) blood pressure compared to NW participants. The systolic (105.20 ± 3.00) as well as diastolic (64.25 ± 1.53) blood pressure levels of the OW group did not show a significant difference with respective NW participants (systolic: 98.22 ± 1.72, diastolic: 63.90 ± 1.52, respectively). The OW and OB group participants did not show any significant difference in heart rate compared to the NW group (Figure 1).

**Figure 2:**
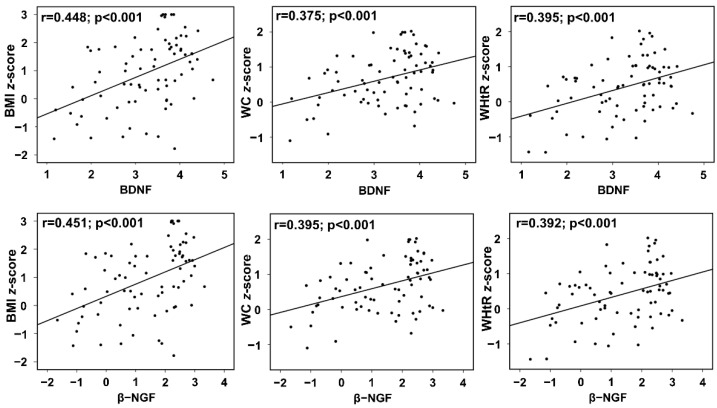
Correlation between anthropometric measurements BMI z-score, WC z-score, and WHtR z-score with neurotrophins BDNF and NGF.

Additionally, the results obtained from unadjusted linear regression analysis to examine the correlation between variables BDNF and β-NGF levels showed a positive association with obesity measures such as BMI z-score, WC z-score, and WHtR z-score. The correlation of BDNF with BMI z-score (r = 0.448, 95% CI: 0.35 to 0.95, *p* < 0.001), WC z-score (r = 0.375, 95% CI: 0.138 to 0.507, *p* < 0.001), and WHtR z-score (r = 0.395, 95% CI: 0.169 to 0.563, *p* < 0.001) was also identified. The analysis was repeated for β-NGF and obesity measures. Similarly, the result of β-NGF linear regression analysis demonstrate a significant correlation with BMI z-score (r = 0.451, 95% CI: 0.234 to 0.632, *p* < 0.001), WC z-score (r = 0.395, 95% CI: 0.105 to 0.347, *p* < 0.001), and WHtR z-score (r = 0.392, 95% CI: 0.110 to 0.372, *p* < 0.001) (Figure 2).

**Figure 3:**
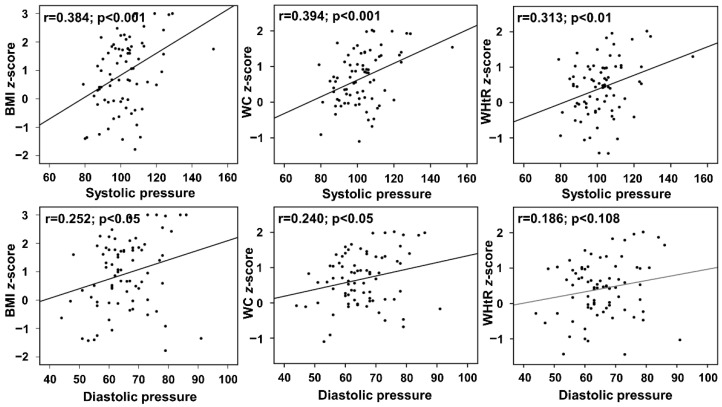
Relation between the anthropometric measurement (BMI z-score, WC z-score, WHtR z-score) with blood pressure (systolic and diastolic) and heart rate.

The results of systolic and diastolic blood pressure association with obesity measures are shown in Figure 3. The results represent a significant positive association of systolic pressure with BMI z-score (r = 0.384, 95% CI: 0.017 to 0.060, *p* < 0.001), WC z-score (r = 0.394, 95% CI: 0.011 to 0.036, *p* < 0.001), and WHtR z-score (r = 0.313, 95% CI: 0.006 to 0.034, *p* < 0.01). The results of diastolic pressure showed significant positive correlation with BMI z-score (r = 0.252, 95% CI: 0.004 to 0.064, *p* < 0.05) and WC z-score (r = 0.240, 95% CI: 0.001 to 0.037, *p* < 0.05), but did not show significant correlation with WHtR z-score (r = 0.186, 95% CI: −0.004 to 0.035, *p* < 0.108). In contrast to systolic pressure, there was no association observed for heart rate when linear regression analysis was applied.

**Figure 4:**
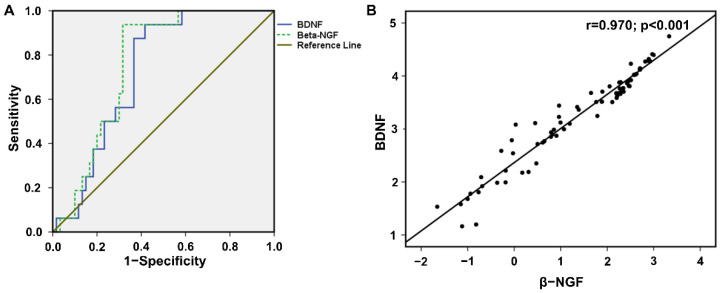
(**A**) The receiver operating characteristics (ROC) curve shows the area under the curve (AUC) and the cutoff value of salivary BDNF and NGF. The diagonal line is the reference line with specificity and sensitivity. (**B**) The scatter plot shows the association of neurotrophins BDNF and β-NGF.

ROC curves were used to analyze the diagnostic efficiency of BDNF and NGF among the study participants. The area under the curve (AUC) results showed β-NGF had a higher AUC (0.757; 95% CI: 0.650–0.864; *p* < 0.005) and cut-off values of 2.08 from the transformed data with a sensitivity of 0.938 and specificity of 0.317. Evaluating the diagnostic performance for early detection of biomarker showed AUC for BDNF (0.727; 95% CI: 0.615–0.839; *p* < 0.002) with sensitivity (0.875), specificity (0.367), and the cut off values (3.551). The diagnostic power of β-NGF and BDNF showed significant AUC values, and this helps for the early detection of obesity and associated risk factors in children (Figure 4A).

**Figure 5:**
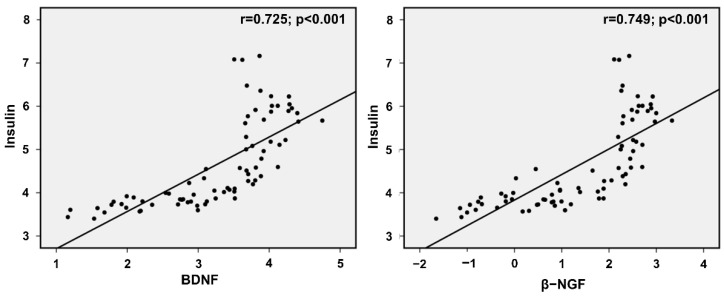
Correlation showed a significant association between insulin and neurotrophins BDNF and NGF. The results show r = 0.725 and r = 0.749, respectively.

To determine the relationship between BDNF and β-NGF in obese children, linear regression analysis was performed. The results of the BDNF and β-NGF association demonstrate a significant positive correlation, with an r-value of 0.970 (95% CI: 0.607–0.681; *p* < 0.001) (Figure 4B). Our previously published data showed a higher expression of insulin in OB group participants compared to the NW group [7]. In this study, we analyzed insulin association with neurotrophic parameters BDNF and NGF to measure insulin dependency in NW and OB participants. The results showed a positive correlation of insulin with BDNF and β-NGF, with a respective r-value of 0.725 (95% CI: 0.671–1.051; *p* < 0.001) and 0.749 (95% CI: 0.470–0.712; *p* < 0.001) (Figure 5).

To test the relationship between socioeconomic status (SES) and health outcomes, the odds ratio was calculated by multinomial regression analysis. The OB group showed significant unadjusted β-coefficient values with reference to the NW weight category for salivary BDNF (OR = 1.036; *p* < 0.011), β-NGF (OR = 1.163; *p* < 0.004) and insulin (OR = 1.006; *p* < 0.002), along with both systolic (OR = 1.085; *p* < 0.005) and diastolic (OR = 1.084; *p* < 0.021) blood pressure. The measures were adjusted for age, gender, income, and maternal education to verify the SES correlation. After adjustment with cofounders, the β-coefficient was shown to be significant in salivary markers and systolic blood pressure. Diastolic pressure did not show as significant after adjusting with cofounders (Table 1).

## 4. Discussion

The study analyzed the BDNF and NGF levels in the saliva of NW, OW, and OB children. The results show that BDNF and NGF concentrations were significantly increased in OB children compared with NW children, which correlates with obesity measures, indicating a correlation between neurotrophic proteins and fat mass. Similarly, systolic and diastolic blood pressure was found to be high in OB participants compared to NW. We could not detect any difference in the heart rate between NW and OW or OB group participants. The current study was designed to evaluate the neurotrophic proteins BDNF and NGF as the salivary marker of obesity in children. Childhood obesity is considered one process that generates low-grade inflammation [32]. Neurons are protected by neurotrophic factors from inflammatory outcomes and play an important role in regulating dietary intake and changes in body weight in children [33]. The results of BDNF and NGF concentrations correlate with the previously published studies. The cross-sectional and longitudinal data analysis performed on normal-weight and obese children showed significantly increased serum BDNF in obese children than in normal-weight children [34]. Plasma BDNF levels in the pediatric population age range of 5–13 years were shown to be significantly elevated in overweight and obese children compared to normal-weight children [35]. Neurotrophin NGF concentration in adult female participants with different degrees of obesity showed a 1.4-fold increase in overweight and obese participants in comparison to normal-weight participants. Plasma NGF was decreased in morbidly obese participants compared to overweight and obese participants, but its level was more elevated than in normal-weight participants [13]. Ha et al. reported that higher circulating CRP and neurotrophin (BDNF and NGF) were observed in obese adolescents. Our result converged with previous findings [36]. The higher amount of BDNF is in accordance with the previous study that the increase could be linked to genetic factors [37].

In the present study, systolic and diastolic blood pressure was higher in obese children compared to overweight and normal-weight groups. Previous findings recorded a 31% elevated blood pressure in overweight and obese children, and in adolescents aged 9–17 years [38]. It has been reported in a study conducted on children and adolescents that there was a significantly higher systolic and diastolic blood pressure in overweight and obese compared to control participants [39]. A higher systolic and diastolic pressure was recorded in obese compared to lean participants in a study including middle-aged and elderly subjects. The significant correlation between diastolic pressure and BDNF in both males and females strongly suggests that plasma BDNF is important for cardiovascular disease [40]. In addition, the study showed a positive association of BDNF with BMI in female participants, but not in males. As shown in previous studies, a significantly positive correlation was observed between obesity measures (BMI, WC z-score, and WHtR z-score) and neurotrophins (BDNF and NGF) in our study. Roth et al. demonstrated a significant positive association of BMI and BDNF and leptin levels. Haploinsufficiency in human BDNF is negatively correlated with obesity, which emphasizes the pivotal role of BDNF in energy homeostasis regulation [41]. A recent report on plasma BDNF levels correlated significantly with WC, BMI, glucose, and systolic pressure among the pediatric population. The results evidence that BDNF levels tend to increase in association with BMI, with an energy homeostasis regulation in obese children [35].

On the other hand, the plasma concentration of NGF showed a significantly positive association with BMI, but the participants did not express a significant association with BDNF [13]. The results describe the relationship between salivary BDNF and NGF concentration, adiposity index, and fat mass in children. Although BMI may be a good indicator for overweight, it is an indirect measure of fat mass. In the current study, salivary BDNF and NGF levels were positively associated with BMI z-score, WC z-score, and WHtR z-score. Concerning blood pressure, a large school-based children’s study conducted in China resulted in a positive correlation of BMI and WC with systolic and diastolic blood pressure [42]. A cross-sectional evaluation of school children showed a gradual elevation of blood pressure observed with increasing waist circumference [43]. A community-based cross-sectional study conducted on the Taiwan study population showed a positive correlation between BMI, WC, and systolic blood pressure [44], and these findings are congruent with our results.

The receiver-operating characteristics curve of BDNF and NGF is a good marker of obesity and metabolic complications with high sensitivity and specificity. In recent clinical study data of healthy and type 2 diabetes patients, diagnostic values of BDNF and NGF showed high sensitivity and specificity with very good AUC values [45]. The ROC curves for obesity prediction on the BDNF of a case-control study on school children expressed a prediction value above 0.65 in two different models [46]. In the current study, BDNF and NGF showed a positive association with insulin, and this result is supported by Levinger et al. A study conducted on middle-aged individuals reported a positive correlation between plasma BDNF and insulin [47]. Pedersen et al. reported in the Childhood Health, Activity, and Motor Performance School Study Denmark (CHAMPS-study DK) that serum BDNF was positively associated with insulin in the healthy adolescent population [48]. We also found a positive correlation between both neurotrophic factors (NGF and BDNF), and this is consistent with the higher expression of NGF and BDNF of overweight and obese participants [13].

## 5. Conclusions

BDNF and NGF levels were higher in obese children compared to normal-weight children, indicating the connection between neurotrophic factors and fat mass. The outcome of the ROC curve had a good diagnostic value of BDNF and NGF, which suggests that they can be used as salivary biomarkers. Both neurotrophic factors have a positive association with insulin and obesity measures. These findings suggest that both BDNF and NGF may play pivotal roles in the pathogenesis of childhood obesity and act as a salivary biomarkers for predicting obesity-related complications.

## Figures and Tables

**Table 1:** Odds ratios of multinominal regression analysis adjusted for age, gender, income, and maternal education (Reference category: NW).

Parameters	Crude β Coefficient (SE)						Adjusted β Coefficient (SE)					
	OW			OB			OW			OB		
	β	OR	*p*	β	OR	*p*	β	OR	*p*	β	OR	*p*
BDNF	0.020 (0.013)	1.020	0.128	0.035 (0.014)	1.036	**0.011**	0.013 (0.014)	1.01	0.35	0.066 (0.022)	1.068	**0.002**
β-NGF	0.066 (0.048)	1.069	0.171	0.151 (0.053)	1.163	**0.004**	0.041 (0.052)	1.04	0.43	0.273 (0.086)	1.314	**0.001**
Insulin	0.004 (0.002)	1.004	0.041	0.006 (0.002)	1.006	**0.002**	0.004 (0.002)	1.004	0.075	0.008 (0.003)	1.008	**0.002**
Systolic blood pressure	0.060 (0.027)	1.062	**0.027**	0.081 (0.029)	1.085	**0.005**	0.105 (0.039)	1.111	**0.007**	0.113 (0.042)	1.120	**0.007**
Diastolic blood pressure	0.005 (0.032)	1.005	0.883	0.081 (0.035)	1.084	**0.021**	0.004 (0.039)	1.004	0.922	0.079 (0.043)	1.082	0.063
Heart rate	0.006 (0.024)	1.006	0.814	−0.014 (0.027)	0.986	0.601	0.007 (0.028)	1.007	0.817	−0.027 (0.032)	0.973	0.405

BDNF—Brain derived neurotrophic factor; β-NGF—β-nerve growth factor; NW—Normal-weight; OW—Overweight; OB—Obese; OR—Odds ratio; SE—Standard error. The *p*-value in bold represents the significant odds ratio of the OW or OB participants compared to the NW group.

## Data Availability

The study datasets of the current manuscript are available from the corresponding author upon request.

## Supplementary Materials

The following supporting information can be downloaded at: https://www.mdpi.com/article/10.3390/diagnostics12051130/s1, Table S1: Anthropometric measurements of study subjects.

## References

[1] Hruby A., Hu F.B. (2015). The Epidemiology of Obesity: A Big Picture. Pharmacoeconomics.

[2] Hu S., Loo J.A., Wong D.T. (2006). Human body fluid proteome analysis. Proteomics.

[3] Centers for Disease Control and Preventio (CDC) Prevalence of Obesity among Adults and Youth: United States, 2015–2016. https://www.cdc.gov/nchs/products/databriefs/db288.htm#:~:text=The%20prevalence%20of%20obesity%20among%20U.S.%20youth%20was%2018.5%25%20in,5%20years)%20(13.9%25.

[4] Mayeux R. (2004). Biomarkers: Potential uses and limitations. NeuroRx.

[5] Myette-Cote E., Baba K., Brar R., Little J.P. (2017). Detection of Salivary Insulin Following Low versus High Carbohydrate Meals in Humans. Nutrients.

[6] Pico C., Serra F., Rodriguez A.M., Keijer J., Palou A. (2019). Biomarkers of Nutrition and Health: New Tools for New Approaches. Nutrients.

[7] Selvaraju V., Babu J.R., Geetha T. (2022). Multiplexed measurements of salivary fetuin-A, insulin, and adiponectin as potential non-invasive biomarkers in childhood obesity. Cytokine.

[8] Srinivasan M., Meadows M.L., Maxwell L. (2018). Assessment of Salivary Adipokines Resistin, Visfatin, and Ghrelin as Type 2 Diabetes Mellitus Biomarkers. Biochem. Res. Int..

[9] Yoshizawa J.M., Schafer C.A., Schafer J.J., Farrell J.J., Paster B.J., Wong D.T. (2013). Salivary biomarkers: Toward future clinical and diagnostic utilities. Clin. Microbiol. Rev..

[10] Chauhan A., Yadav S.S., Dwivedi P., Lal N., Usman K., Khattri S. (2016). Correlation of Serum and Salivary Cytokines Level With Clinical Parameters in Metabolic Syndrome With Periodontitis. J. Clin. Lab. Anal..

[11] Ladgotra A., Verma P., Raj S.S. (2016). Estimation of Salivary and Serum Biomarkers in Diabetic and Non Diabetic Patients—A Comparative Study. J. Clin. Diagn. Res..

[12] Rosas-Vargas H., Martinez-Ezquerro J.D., Bienvenu T. (2011). Brain-derived neurotrophic factor, food intake regulation, and obesity. Arch. Med. Res..

[13] Bullo M., Peeraully M.R., Trayhurn P., Folch J., Salas-Salvado J. (2007). Circulating nerve growth factor levels in relation to obesity and the metabolic syndrome in women. Eur. J. Endocrinol..

[14] Lebrun B., Bariohay B., Moyse E., Jean A. (2006). Brain-derived neurotrophic factor (BDNF) and food intake regulation: A minireview. Auton. Neurosci..

[15] Rios M. (2013). BDNF and the central control of feeding: Accidental bystander or essential player?. Trends Neurosci..

[16] Saito S., Watanabe K., Hashimoto E., Saito T. (2009). Low serum BDNF and food intake regulation: A possible new explanation of the pathophysiology of eating disorders. Prog. Neuropsychopharmacol. Biol. Psychiatry.

[17] Krabbe K.S., Nielsen A.R., Krogh-Madsen R., Plomgaard P., Rasmussen P., Erikstrup C., Fischer C.P., Lindegaard B., Petersen A.M., Taudorf S. (2007). Brain-derived neurotrophic factor (BDNF) and type 2 diabetes. Diabetologia.

[18] Lommatzsch M., Zingler D., Schuhbaeck K., Schloetcke K., Zingler C., Schuff-Werner P., Virchow J.C. (2005). The impact of age, weight and gender on BDNF levels in human platelets and plasma. Neurobiol. Aging.

[19] Alomari M.A., Khabour O.F., Alawneh K., Alzoubi K.H., Maikano A.B. (2020). The importance of physical fitness for the relationship of BDNF with obesity measures in young normal-weight adults. Heliyon.

[20] Glud M., Christiansen T., Larsen L.H., Richelsen B., Bruun J.M. (2019). Changes in Circulating BDNF in relation to Sex, Diet, and Exercise: A 12-Week Randomized Controlled Study in Overweight and Obese Participants. J. Obes..

[21] Katuri R.B., Gaur G.S., Sahoo J.P., Bobby Z., Shanmugavel K. (2021). Association of Circulating Brain-Derived Neurotrophic Factor with Cognition among Adult Obese Population. J. Obes. Metab. Syndr..

[22] Sandrini L., Di Minno A., Amadio P., Ieraci A., Tremoli E., Barbieri S.S. (2018). Association between Obesity and Circulating Brain-Derived Neurotrophic Factor (BDNF) Levels: Systematic Review of Literature and Meta-Analysis. Int. J. Mol. Sci..

[23] Lee I.T., Wang J.S., Fu C.P., Lin S.Y., Sheu W.H. (2016). Relationship between body weight and the increment in serum brain-derived neurotrophic factor after oral glucose challenge in men with obesity and metabolic syndrome: A prospective study. Medicine.

[24] Ginsberg H.N., MacCallum P.R. (2009). The obesity, metabolic syndrome, and type 2 diabetes mellitus pandemic: Part I. Increased cardiovascular disease risk and the importance of atherogenic dyslipidemia in persons with the metabolic syndrome and type 2 diabetes mellitus. J. Cardiometab. Syndr..

[25] Borelli V., Marchioli A., Di Taranto R., Romano M., Chiandussi S., Di Lenarda R., Biasotto M., Zabucchi G. (2010). Neuropeptides in saliva of subjects with burning mouth syndrome: A pilot study. Oral Dis..

[26] Jang M.U., Park J.W., Kho H.S., Chung S.C., Chung J.W. (2011). Plasma and saliva levels of nerve growth factor and neuropeptides in chronic migraine patients. Oral Dis..

[27] Saruta J., Sato S., Tsukinoki K. (2010). The role of neurotrophins related to stress in saliva and salivary glands. Histol. Histopathol..

[28] Selvaraju V., Babu J.R., Geetha T. (2019). Association of salivary C-reactive protein with the obesity measures and markers in children. Diabetes Metab. Syndr. Obes..

[29] Kuczmarski R.J., Ogden C.L., Guo S.S., Grummer-Strawn L.M., Flegal K.M., Mei Z., Wei R., Curtin L.R., Roche A.F., Johnson C.L. (2002). 2000 CDC Growth Charts for the United States: Methods and Development.

[30] Butte N.F., Garza C., de Onis M. (2007). Evaluation of the feasibility of international growth standards for school-aged children and adolescents. J. Nutr..

[31] Sharma A.K., Metzger D.L., Daymont C., Hadjiyannakis S., Rodd C.J. (2015). LMS tables for waist-circumference and waist-height ratio Z-scores in children aged 5-19 y in NHANES III: Association with cardio-metabolic risks. Pediatr. Res..

[32] Vachharajani V., Granger D.N. (2009). Adipose tissue: A motor for the inflammation associated with obesity. IUBMB Life.

[33] El-Alameey I.R., Ahmed H.H., Abushady M.M. (2019). Role of Lifestyle Intervention Program in Regulating Brain Derived Neurotrophic Factor in Obese Children with Metabolic Syndrome Components. Biomed. Pharmacol. J..

[34] Roth C.L., Elfers C., Gebhardt U., Muller H.L., Reinehr T. (2013). Brain-derived neurotrophic factor and its relation to leptin in obese children before and after weight loss. Metabolism.

[35] Villalobos Gutierrez P.T., Delgado G.G., Renteria C.T., Orozco E.R., Coronado O.G. (2020). Obesity and Overweight Influence BDNF Serum Levels in the Pediatric Population. Metab.—Clin. Exp..

[36] Ha J., Cohen J.I., Tirsi A., Convit A. (2013). Association of obesity-mediated insulin resistance and hypothalamic volumes: Possible sex differences. Dis. Markers.

[37] Noble E.E., Billington C.J., Kotz C.M., Wang C. (2011). The lighter side of BDNF. Am. J. Physiol. Regul. Integr. Comp. Physiol..

[38] Mazor-Aronovitch K., Lotan D., Modan-Moses D., Fradkin A., Pinhas-Hamiel O. (2014). Blood pressure in obese and overweight children and adolescents. Isr. Med. Assoc. J..

[39] Schiel R., Beltschikow W., Kramer G., Stein G. (2006). Overweight, obesity and elevated blood pressure in children and adolescents. Eur. J. Med. Res..

[40] Golden E., Emiliano A., Maudsley S., Windham B.G., Carlson O.D., Egan J.M., Driscoll I., Ferrucci L., Martin B., Mattson M.P. (2010). Circulating brain-derived neurotrophic factor and indices of metabolic and cardiovascular health: Data from the Baltimore Longitudinal Study of Aging. PLoS ONE.

[41] Han J.C., Liu Q.R., Jones M., Levinn R.L., Menzie C.M., Jefferson-George K.S., Adler-Wailes D.C., Sanford E.L., Lacbawan F.L., Uhl G.R. (2008). Brain-derived neurotrophic factor and obesity in the WAGR syndrome. N. Engl. J. Med..

[42] Lu X., Shi P., Luo C.Y., Zhou Y.F., Yu H.T., Guo C.Y., Wu F. (2013). Prevalence of hypertension in overweight and obese children from a large school-based population in Shanghai, China. BMC Public Health.

[43] Pazin D.C., Rosaneli C.F., Olandoski M., Oliveira E.R.N., Baena C.P., Figueredo A.S., Baraniuk A.O., Kaestner T., Guarita-Souza L.C., Faria-Neto J.R. (2017). Waist Circumference is Associated with Blood Pressure in Children with Normal Body Mass Index: A Cross-Sectional Analysis of 3417 School Children. Arq. Bras. Cardiol..

[44] Lin Y.A., Chen Y.J., Tsao Y.C., Yeh W.C., Li W.C., Tzeng I.S., Chen J.Y. (2019). Relationship between obesity indices and hypertension among middle-aged and elderly populations in Taiwan: A community-based, cross-sectional study. BMJ Open.

[45] Sun Q., Tang D.D., Yin E.G., Wei L.L., Chen P., Deng S.P., Tu L.L. (2018). Diagnostic Significance of Serum Levels of Nerve Growth Factor and Brain Derived Neurotrophic Factor in Diabetic Peripheral Neuropathy. Med. Sci. Monit..

[46] Tuyet L.T., Nhung B.T., Dao D.T.A., Hanh N.T.H., Tuyen L.D., Binh T.Q., Thuc V.T.M. (2017). The Brain-Derived Neurotrophic Factor Val66Met Polymorphism, Delivery Method, Birth Weight, and Night Sleep Duration as Determinants of Obesity in Vietnamese Children of Primary School Age. Child Obes..

[47] Levinger I., Goodman C., Matthews V., Hare D.L., Jerums G., Garnham A., Selig S. (2008). BDNF, metabolic risk factors, and resistance training in middle-aged individuals. Med. Sci. Sports Exerc..

[48] Pedersen N.H., Tarp J., Andersen L.B., Gejl A.K., Huang T., Peijs L., Bugge A. (2017). The association between serum brain-derived neurotrophic factor and a cluster of cardiovascular risk factors in adolescents: The CHAMPS-study DK. PLoS ONE.

