# Supplementary material for: Salivary Neurotrophins Brain-Derived Neurotrophic Factor and Nerve Growth Factor Associated with Childhood Obesity: A Multiplex Magnetic Luminescence Analysis
Source: Diagnostics (Basel). 2022 May 3;12(5):1130. doi: 10.3390/diagnostics12051130 (PMC9140051; doi:10.3390/diagnostics12051130)
Supplement: Supplementary file 1 [file diagnostics-12-01130-s001.zip › diagnostics-1711912-supplementary.pdf]

Table S1. Anthropometric measurements of study subjects

Parameter		All	NW	OW	OB
**Number of Participants**		76	40 (52.6%)	20 (26.3%)	16 (21.1%)
**Sex**	**Male**	41	20 (48.8%)	14 (34.1%)	7 (17.1%)
	**Female**	35	20 (57.1%)	6 (17.1%)	9 (25.7%)
**Age (years)**		8.43 ± 0.16	8.38 ± 0.23	8.32 ± 0.29	8.70 ± 0.37
**BMI (kg/m^2)**		18.42 ± 0.40	16.01 ± 0.24	19.29 ± 0.22	23.36 ± 0.80
**BMI *z*-score**		0.94 ± 0.15	-0.04 ± 0.13	1.57 ± 0.07	2.58 ± 0.13
**Waist circumference (cm)**		65.39 ± 1.01	60.18 ± 0.74	66.65 ± 1.11	76.87 ± 2.26
**WC *z*-score**		0.68 ± 0.08	0.15 ± 0.08	0.98 ± 0.36	1.60 ± 0.09
**WHtR *z*-score**		0.42 ± 0.09	-0.13 ± 0.10	0.73 ± 0.06	1.37 ± 0.11

Results are expressed as Mean ± SEM. The data was presented in our previously published article [7], and this table was reproduced with Journal's permission.

Abbreviation: BMI – body mass index; WC – waist circumference; WHtR – waist-to-height ratio; NW – normal weight; OW – overweight; OB – obese.
